# Dosimetric comparison between VMAT plans using the fast-rotating O-ring linac with dual-layer stacked MLC and helical tomotherapy for nasopharyngeal carcinoma

**DOI:** 10.1186/s13014-022-02124-0

**Published:** 2022-09-12

**Authors:** Sang Gyu Ju, Yong Chan Ahn, Yeong-bi Kim, Jin Man Kim, Dong Yeol Kwon, Byoung Suk Park, Kyungmi Yang

**Affiliations:** Department of Radiation Oncology, Samsung Medical Center, Sungkyunkwan University School of Medicine, Irwon-Ro 81, Gangnam-Gu, Seoul, 06351 Republic of Korea

**Keywords:** Nasopharyngeal cancer, Halcyon, Tomotherapy, Plan comparison, VMAT, IMRT

## Abstract

**Background:**

To compare the dosimetric profiles of volumetric modulated arc therapy (VMAT) plans using the fast-rotating O-ring linac (the Halcyon system) based on a dual-layer stacked multi-leaf collimator and helical tomotherapy (HT) for nasopharyngeal cancer (NPCa).

**Methods:**

For 30 NPCa patients, three sets of RT plans were generated, under the same policy of contouring and dose constraints: HT plan; Halcyon VMAT plan with two arcs (HL_2arc_); and Halcyon VMAT plan with four arcs (HL_4arc_), respectively. The intended dose schedule was to deliver 67.2 Gy to the planning gross target volume (P-GTV) and 56.0 Gy to the planning clinical target volume (P-CTV) in 28 fractions using the simultaneously integrated boost concept. Target volumes and organ at risks dose metrics were evaluated for all plans. Normal tissue complication probabilities (NTCP) for esophagus, parotid glands, spinal cord, and brain stem were compared.

**Results:**

The HT plan achieved the best dose homogeneity index for both P_GTV and P_CTV, followed by the HL_4arc_ and L_2arc_ plans. No significant difference in the dose conformity index (CI) for P_GTV was observed between the HT plan (0.80) and either the HL_2arc_ plan (0.79) or the HL_4arc_ plan (0.83). The HL_4arc_ plan showed the best CI for P_CTV (0.88), followed by the HL_2arc_ plan (0.83) and the HT plan (0.80). The HL_4arc_ plan (median, interquartile rage (Q1, Q3): 25.36 (22.22, 26.89) Gy) showed the lowest D_mean_ in the parotid glands, followed by the HT (25.88 (23.87, 27.87) Gy) and HL_2arc_ plans (28.00 (23.24, 33.99) Gy). In the oral cavity (OC) dose comparison, the HT (22.03 (19.79, 24.85) Gy) plan showed the lowest D_mean_ compared to the HL_2arc_ (23.96 (20.84, 28.02) Gy) and HL_4arc_ (24.14 (20.17, 27.53) Gy) plans. Intermediate and low dose regions (40–65% of the prescribed dose) were well fit to the target volume in HL_4arc_, compared to the HT and HL_2arc_ plans. All plans met the dose constraints for the other OARs with sufficient dose margins. The between-group differences in the median NTCP values for the parotid glands and OC were < 3.47% and < 1.7% points, respectively.

**Conclusions:**

The dosimetric profiles of Halcyon VMAT plans were comparable to that of HT, and HL_4arc_ showed better dosimetric profiles than HL_2arc_ for NPCa.

**Supplementary Information:**

The online version contains supplementary material available at 10.1186/s13014-022-02124-0.

## Background

Radiation therapy (RT) has long been the primary treatment modality in treating the patients with nasopharynx cancer (NPCa) [1–4]. RT for NPCa is, however, sometimes challenging as the target volumes are frequently large, deep-seated, and close to or even overlapping with the surrounding organs at risk (OARs). Several types of acute and delayed radiation-induced toxicities, including xerostomia, oral mucositis, esophagitis, myelitis, and brain stem necrosis, are inevitably accompanied during and after high dose RT depending on the radiation dose level and anatomical proximity among the radiation targets and surrounding OARs.

In an effort to reduce radiation-induced toxicities, advanced RT techniques which can provide highly conformal dose to the target volume, such as intensity modulated RT (IMRT), have been increasingly applied in treatment of the NPCa patients. In particular, IMRT has contributed to lowering radiation myelitis and brain stem necrosis by reducing the dose to the spinal cord and brain stem, which were rather frequently encountered during the conventional RT technique era [5]. Radiation-induced oral mucositis and xerostomia, however, still remain the annoying prices to be paid by most patients during the dose escalation IMRT for the NPCa patients [4, 6–9]. Furthermore, most normal tissues in the head and neck (the non-target volume) are sensitive to the radiation damage, so the reduction of the normal tissue dose as much as possible is still critical in reducing radiation-induced toxicity and improving the quality of life during and after RT [4, 6].

To meet these clinical needs, the development of new treatment techniques based on advanced treatment devices has been in progress continuously. The quality of the IMRT plan is closely related to many technical parameters: the characteristics of the multi-leaf collimator (MLC); inverse dose optimization algorithm; dose calculation engine, and beam delivery technique including treatment machine capability, respectively. There have been several technological advances in this regards, and volumetric modulated arc therapy (VMAT) is known to provide better dose distribution than static IMRT technique, and has become the mainstream IMRT beam delivery option in treating various cancer types including NPCa [10, 11].

The characteristics of MLC have an influence on dosimetric profiles of VMAT plan because they play an important role in both beam shaping and intensity modulation along with dose optimization algorithm. Particularly, the width and transmission of the MLC directly affect the target dose conformity and normal organ dose outside the target, respectively. The recently introduced fast-rotating O-ring linac (FOL), Halcyon system (Varian Medical Systems, Palo Alto, CA, USA) employed the unique staggered DL-MLC by using two MLCs of 1 cm width (actual resolution 0.5 cm) [12]. The DL-MLC can produce low MLC transmission (0.01% of the primary beam), and minimal tongue and groove effect when compared with the traditional C-arm Linac (1.36%) [12–14] and helical tomotherapy (HT, 0.53% interleaf leakage) [15]. In contrast, HT has binary MLCs (0.625 cm width), but it can generate fine optimization resolution (of a few millimeters), combined with varying jaw width and pitch [16]. Furthermore, HT employs a helical beam delivery with the dynamic jaw technique to enhance dose conformity to the target volume in the inferior-superior border of the target volume while minimizing the OAR dose [17], which can generate better dosimeric profiles in RT for NPCa [18–21]. These differences are expected to act differently in the treatment plan for NPCa, but sufficient studies have not been reported.

Understanding the capabilities of the new techniques, which are closely related to the OARs’ dose sparing, is important in selecting the treatment techniques for better clinical outcomes. Several studies have assessed the FOL plan in relation to the C-arm Linac plan in various treatment sites [10–20]. However, there are few plan comparison studies between the FOL and HT [22, 23], and there have been no reports of dose comparison studies for NPCa. Therefore, we performed the first comparative analysis of the dosimeric profiles between VMAT plans using FOL with DL-MLC and HT for NPCa.

## Methods and materials

### Patient selection and simulation

This dosimetric comparison study did not involve any experiments on humans or animals. With the approval from the institutional review board (IRB SMC 2020-08-120-001), 30 consecutive NPCa patients, who underwent definitive RT based on concurrent chemotherapy between May 2018 and April 2019 with HT (TomoHD™, Accuray®, Sunnyvale, CA, USA) at the authors’ institute were included in this study (Table 1). All patients underwent planning computed tomography (CT) (2.5-mm slice thickness; Discovery RT590, GE Healthcare, Milwaukee, WI) in the supine position with a customized immobilization device (thermoplastic mask, Aquaplast RT™, Q-fix®, USA) and a tongue immobilization device [24].

**Table 1:** Patients' characteristics

Characteristics	Total (N = 30)
Median age (range)	51.5 (30–72) years
Sex	
Female	7 (25.0%)
Male	23 (75.0%)
Histology	
Non-keratinizing squamous cell carcinoma	25 (83.3%)
Undifferentiated carcinoma	5 (16.7%)
T stage	
cT1	18 (60.0%)
cT2	1 (3.3%)
cT3	9 (30.0%)
cT4	2 (6.7%)
N stage	
cN0	3 (10.0%)
cN1	9 (30.0%)
cN2	11 (36.7%)
cN3	7 (23.3%)
AJCC stage (8th Ed)	
I	1 (3.3%)
II	5 (16.7%)
III	15 (50.0%)
Iva	9 (30.0%)

The gross tumor volume (GTV) and clinical target volume (CTV) were delineated based on all available clinical information, including diagnostic images. The planning target volumes (PTVs) were generated by adding 3-mm isotropic expansions from the GTV (P-GTV) and CTV (P-CTV), which were edited considering the actual anatomic boundaries, such as the spinal cord and skin surface. The OARs, including the spinal cord, brainstem, parotid gland, esophagus, optic nerve, optic chiasm, submandibular gland (SMG), cochlea, and OC were delineated according to previously published guidelines [9, 25]. The planning risk volumes were generated by adding 3-mm isotropic expansions for the spinal cord (P-cord) and optic apparatus. Delineation of all contours was performed by one radiation oncologist to ensure consistency.

### Treatment planning

The HT plan (Precision™, Version 1.1.1.1, Accuray®, Sunnyvale, USA) and the Halcyon VMAT plans (Eclipse, Version 15.6, Varian Medical systems, Palo Alto, USA) with two arcs (HL_2arc_) and four arcs (HL_4arc_) were generated using the same planning CT and contours for the target and OARs with a 6-MV flattening-filter-free (FFF) beam. The typical dose schedule was 67.2 Gy to the P-GTV and 56.0 Gy to the P-CTV in 28 fractions using the simultaneously integrated boost.

The same dose constraints based on the internal plan guideline were applied to all plans (Table 2). Four constraints were set at the highest priority level for the PTVs, P-cord (maximum dose [D_max_] ≤ 45 Gy), optic nerve, and optic chiasm (D_max_ ≤ 50 Gy). The second priority level was given to the brain stem (D_max_ ≤ 50 Gy), parotid gland (mean dose [D_mean_] ≤ 26 Gy), and OC (D_mean_ ≤ 30 Gy). The third priority level was given to the planning lens (P_lens) (D_max_ ≤ 10 Gy), eyeball (D_max_ ≤ 50 Gy), and esophagus and cochlea (D_mean_ ≤ 35 Gy). The lowest level constraint was given to the SMG (D_mean_ ≤ 30 Gy) and brain (D_max_ ≤ 60 Gy).

**Table 2:** Dose constraints for inverse planning

Priority	Structure	Constraints
1	P-GTV	D_95_ ≥ 67.2 Gy (100%) or D_99_ ≥ 95% of the prescribed dose (67.2 Gy)
		V_73.9 (110%)_ ≤ 1 cm^3^
	P-CTV	D_95_ ≥ 56.0 Gy (100%) or D_98_ ≥ 95% of the prescribed dose (56.0 Gy (100%))
	P-cord^a^	D_max_ ≤ 45.0 Gy
	Optic nerve and optic chiasm	D_max_ ≤ 50.0 Gy
2	Brain stem	D_max_ ≤ 50.0 Gy
	Parotid glands	D_mean_ ≤ 26.0 Gy
	Oral cavity	D_mean_ ≤ 30.0 Gy
		V_30_ ≤ 30%
3	Lens	D_max_ ≤ 10.0 Gy
	Eyeball	D_max_ ≤ 50.0 Gy
	Esophagus and Cochlea	D_mean_ ≤ 35.0 Gy
4	Submandibular glands	D_mean_ ≤ 30.0 Gy
	Brain	D_max_ ≤ 60.0 Gy

P-GTV, planning gross target volume; P-CTV, planning clinical target volume; P-cord, planning spinal cord; D_V_, D dose delivered to V% of organ volume; V_D_, absolute or percentage of organ volume receiving D Gy or higher; D_max_, maximum dose; D_mean_, mean dose

^a^P-Cord means the planning volume for the spinal cord which was generated by adding 3–5 mm margin to the actual spinal cord

For the HT plan, fine plan conditions were used, including a field width of 2.5 cm, modulation factor of 2.0, and pitch of 0.287 to avoid the thread effect [26]. Dynamic jaw mode (TomoEDGE™, Accuray) was employed to improve the longitudinal dose conformity by reducing the penumbra at the inferior and superior borders of the PTV [27]. The final dose was calculated using the collapsed-cone convolution algorithm with a fine dose calculation resolution (0.98 mm in the x–y plane and 2.5 mm in z).

HL_2arc_ and HL_4arc_ plans were created using two and four full dynamic arcs with DL-MLC, respectively. One isocenter with an automatic collimator angle option was employed for both plans. The final dose was calculated using the anisotropic analytical algorithm with fine-dose resolution (0.25 cm) [13].

HT and VMAT plans were generated by dosimetrists specializing in each plan under blind conditions. The plans shared the planning CT image, contour, and plan constraints with the optimization strategy. The same dose optimization strategy was applied based on the same plan conditions as follows. For all plans, the same order of the dose optimization priority was applied by controlling “importance (tomotherapy plan)” and “priority (Eclipse plan)” based on internal guideline (Table 2). Once the PTV dose met the goal, optimization was continued to reduce the doses to the OARs as much as possible while maintaining the PTV dose coverage.

### Dosimetric comparison of the HT, HL_2arc_, and HL_4arc_ plans

All planning data including calculated dose and contour information with CT image set of three VMAT plans on each patient were transferred to MIM Maestro® (MIM Software Inc., USA) using the Digital Imaging and Communications in Medicine protocol, and quantitative analysis of the dose and volume parameters was performed. To evaluate the target dose coverage, the D_max_ received by 2% (D_2_) and the minimum dose received by 98% (D_98_) of the P_GTV and P_CTV, respectively, were compared among the three plans. The homogeneity index (HI = D_5_/D_98_) [28] and conformity index (CI) [29] were also compared.

To evaluate the dose to normal tissues, the following OAR-related dosimetric parameters were compared (Table 3): the D_mean_ to the parotid, esophagus, cochlea, eyeball, and OC; the D_max_ to the brain stem, esophagus, P-cord, optic chiasm, optic nerve, SMG, eyeball, P-lens, and OC; and the volume of the OC that received doses of 15 Gy (V_15_), 30 Gy (V_30_), 45 Gy (V_45_), or more.

**Table 3:** Comparison of dosimetric characteristics

Parameters		HT	HL_2arc_	HL_4arc_	*P*^a^		
		Median (IQR)			HT vs. HL_2arc_	HT vs. HL_4arc_	HL_2arc_ vs. HL_4arc_
P_GTV	D_2_ (Gy)	69.6 (69.30, 70.03)	70.86 (70.13, 71.60)	70.41 (69.63, 70.85)	0.00	0.01	0.00
	D_98_ (Gy)	66.67 (66.51, 66.75)	66.45 (66.37, 66.61)	66.52 (66.41, 66.77)	0.02	0.59	0.15
	HI	1.04 (1.03, 1.05)	1.06 (1.05, 1.08)	1.06 (1.04, 1.06)	0.00	0.00	0.00
	CI	0.80 (0.76, 0.83)	0.79 (0.74, 0.84)	0.83 (0.79, 0.87)	0.56	0.11	0.00
P_CTV	D_2_ (Gy)	69.13 (68.61, 69.48)	69.89 (69.54, 71.14)	69.67 (69.09, 70.24)	0.00	0.00	0.00
	D_98_ (Gy)	55.32 (54.97, 55.69)	55.04 (54.53, 55.85)	55.17 (54.60, 55.68)	0.29	0.98	> 0.99
	HI	1.25 (1.24, 1.26)	1.28 (1.26, 1.29)	1.27 (1.25, 1.28)	0.00	0.01	0.00
	CI	0.80 (0.78, 0.82)	0.83 (0.79, 0.88)	0.88 (0.85, 0.89)	0.03	0.00	0.00
Brain stem	D_max_ (Gy)	46.99 (39.98, 53.64)	39.29 (34.42, 48.36)	39.49 (31.92, 45.92)	0.00	0.00	0.33
p_cord	D_max_ (Gy)	39.91 (31.19, 43.46)	33.38 (29.75, 44.01)	28.32 (25.20, 34.19)	> 0.99	0.00	0.00
Parotid glands	D_mean_ (Gy)	25.88 (23.87, 27.87)	28.00 (23.24, 33.99)	25.36 (22.22, 26.89)	0.04	0.05	0.00
Esophagus	D_mean_ (Gy)	2.00 (0.68, 4.20)	2.97 (0.89, 7.55)	3.38 (1.01, 6.79)	0.00	0.00	> 0.99
Optic chiasm	D_max_ (Gy)	9.58 (5.95, 24.87)	6.07 (4.96, 18.89)	6.22 (4.92, 15.28)	0.00	0.00	0.05
Optic nerve	D_max_ (Gy)	14.03 (7.02, 27.02)	7.85 (5.55, 17.15)	7.47 (5.46, 14.65)	0.00	0.00	0.08
Cochlea	D_mean_ (Gy)	26.05 (22.69, 30.10)	32.97 (29.01, 38.71)	37.40 (33.49, 40.65)	0.00	0.00	0.02
Eyeball	D_max_ (Gy)	14.14 (9.60, 17.42)	11.31 (5.09, 17.74)	10.90 (5.36, 17.50)	0.76	0.14	0.26
Lens	D_max_ (Gy)	2.66 (1.94, 3.95)	3.07 (2.12, 6.05)	3.03 (2.11, 5.36)	0.01	0.00	> 0.99
Oral cavity	D_mean_ (Gy)	22.03 (19.79, 24.85)	23.96 (20.84, 28.02)	24.14 (20.17, 27.53)	0.03	0.04	> 0.99
	V15 (%)	63.57 (56.46, 75.79)	86.46 (68.99, 93.65)	82.62 (60.65, 97.29)	0.01	0.00	0.66
	V30 (%)	22.09 (17.03, 29.39)	26.14 (16.58, 38.12)	22.41 (16.04, 32.76)	0.29	> 0.99	0.31
	V45 (%)	6.29 (4.34, 9.73)	5.53 (3.04, 9.33)	6.01 (3.08, 8.30)	0.87	0.01	0.71
SMG	D_mean_ (Gy)	23.49 (21.56, 25.07)	24.01 (22.96, 26.28)	24.17 (22.49, 25.30)	0.02	0.11	0.66
Dose spillage volume	V_HS_ for P_GTV	1.68 (1.35, 2.12)	2.03 (1.40, 3.01)	1.81 (1.22, 2.64)	0.01	> 0.99	0.00
	V_HS_ for P_CTV	0.61 (0.56, 0.71)	0.43 (0.39, 0.55)	0.40 (0.36, 0.42)	0.00	0.00	0.00
	V_IS_ for P_CTV	3.75 (3.40, 4.24)	2.79 (2.56, 3.10)	2.62 (2.46, 2.85)	0.00	0.00	0.03
	V_LS_ for P_CTV	13.02 (11.02, 13.95)	6.14 (5.87, 6.61)	6.00 (5.67, 6.58)	0.00	0.00	0.15

HT, helical tomotherapy; HL_2arc_, halcyon two arc; HL_4arc_, halcyon four arc; P-GTV, planning gross target volume; P-CTV, planning clinical target volume; P-cord, planning spinal cord; SMG, submandibular glans; D_V_, D dose delivered to V% of organ volume; V_D_, percentage of organ volume receiving D Gy or higher; D_max_, maximum dose; D_mean_, mean dose; CI, dose conformity index; HI, dose homogeneity index; V_HS_, high dose-spillage volume; V_IS_, intermediate dose-spillage volume; V_LS_, low dose-spillage volume; IQR, interquartile range (Q1, Q3)

^a^The Wilcoxon signed rank test was used by the Bonferroni correction for multiple testing

Furthermore, dose-spillage volumes (DSVs) were calculated to assess the rapid dose fall-off ability near the PTV according to the treatment technique [30–32]. The DSV was calculated as follows:

1 $${\text{DSV}} = \frac{{V_{X\% } - PTV}}{PTV}$$

where V_X%_ is the volume covered by the X% isodose surface. High DSV (V_HS_) for the P_GTV and P_CTV and intermediate DSV (V_IS_) and low DSV (V_LS_) for the P_CTV were calculated by taking into account the volumes that received ≥ 90%, ≥ 50%, and ≥ 25% of the prescribed dose, respectively [30–32]. The ideal value of the DSV is close to zero, which indicates that the volume that received the prescribed dose fit the shape of the target volume well.

To estimate the impact of dose differences on clinical complications, normal tissue complications (NTCPs), including clinical stricture/perforation of esophagus, xerostomia (parotid), oral mucositis (OC), myelitis (spinal cord), and brain stem necrosis, were calculated using the Poisson-LQ model [33, 34] for all plans. The parameters used for NTCP calculation were taken from a previous study (Additional file 1: Table 1).

For statistical analysis of the dosimetric and NTCP comparisons between the three plans, pairwise comparisons were performed using the Wilcoxon signed-rank test based on the Bonferroni correction (SPSS statistics, version 27, IBM®). A probability level with a *p* value < 0.05 was considered significant.

## Results

### Treatment plan comparison

The comparisons of dosimetric parameters using the three VMAT techniques are summarized in Table 3. In comparison of target dose coverage, D_2_ (maximum dose) of the P_GTV and P_CTV was closest to the prescribed dose in the HT plan, followed by the HL_4arc_ and HL_2arc_ plans (*p* < 0.05). For the D_98_ (minimum dose) for P_GTV, no significant differences were observed between the HT and HL_4arc_, and HL_2arc_ and HL_4arc_ in the pairwise comparisons (*p* > 0.05), however, there was significant difference between the HT and HL_2arc_ plans (*p* < 0.05). There was no significant difference in the D_98_ (minimum dose) for P_CTV between the three plans (*p* > 0.05). Therefore, the HT plan achieved the best HI for both P_GTV and P_CTV, followed by the HL_4arc_ and HL_2arc_ plans (*p* < 0.05).

No significant difference in the CI for P_GTV was observed between the HT plan (0.80) and either the HL_2arc_ plan (0.79) or the HL_4arc_ plan (0.83) (*p* > 0.05), although the HL_4arc_ plan showed superiority over the HL_2arc_ plan in the pairwise comparison (*p* < 0.05). However, the HL_4arc_ plan showed the best CI for P_CTV (0.88), followed by the HL_2arc_ plan (0.83) and the HT plan (0.80) (*p* < 0.05).

Although the HL_4arc_ plan showed the lowest D_max_ in the P_cord, optic nerve, and eyeball, followed by the HL_2arc_ and HT plans, all plans met the dose constraints with sufficient dose margins. Furthermore, both HL_2arc_ and HL_4arc_ plans showed lower D_max_ in the brain stem and optic chiasm compared to the HL plan (*p* < 0.05). In contrast, the HT plan showed a lower D_max_ in the lens and a lower D_mean_ in the SMG, cochlea and esophagus than the HL_2arc_ and HL_4arc_ plans. However, all values were within the dose constraints. The HL_4arc_ (median, interquartile range [IQR] (Q1, Q3): 25.36 (22.22, 26.89) Gy) and HT (25.88 (IQR: 23.87, 27.87) Gy) plans showed lower D_mean_ in the parotid gland compared to the HL_2arc_ plan (28.00 (IQR: 23.24, 33.99) Gy) (*p* < 0.05). In OC dose comparison, the HT (22.03 (IQR: 19.79, 24.85) Gy and 63.57 (IQR: 56.46, 75.79)%) plan showed the lowest D_mean_ and V_15_ compared to the HL_2arc_ (23.96 (IQR: 20.84, 28.02) Gy and 86.46 (IQR: 68.99, 93.65)%) and HL_4arc_ (24.14 (IQR: 20.17, 27.53) Gy and 82.62 (IQR: 60.65, 97.29)%) plans (*p* < 0.05), whereas there was no significant difference in V_30_ between the plans (*p* > 0.05). However, the HL_4arc_ (6.01 (IQR: 3.08, 8.30)%) plan had a lower V_45_ than the HT (6.29 (IQR: 4.34, 9.73)%) plan.

**Fig. 1:**
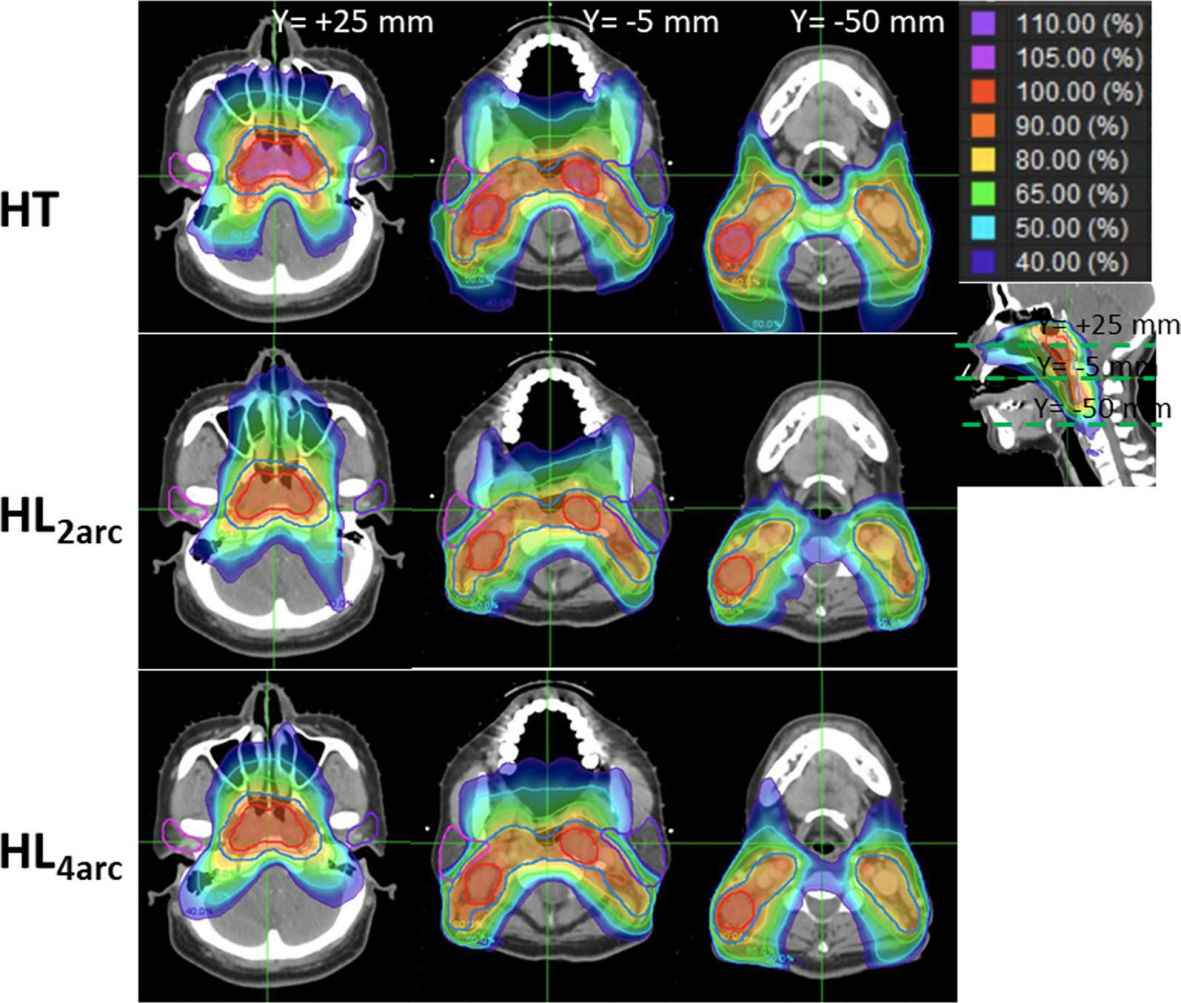
Comparison of axial dose distribution for a nasopharyngeal cancer patient, helical tomotherapy (HT, upper), two-arc halcyon (HL_2arc_, middle), and four-arc halcyon (HL_4arc_, low) plans. Intermediate- and low dose regions (40–65% of the prescribed dose) were well fit to target volume in HL_4arc_ compared to HT and HL_2arc_ plans

For DSV analysis, the HT (1.68 (IQR: 1.35, 2.12) plan had the lowest median V_HS_ for P_GTV, followed by the HL_4arc_ (1.81 (IQR: 1.22, 2.64) and HL_2arc_ (2.03 (IQR:1.40, 3.01) plans. No significant difference was observed between the HT and HL_4arc_ plans in pairwise comparison (*p* > 0.05), although the V_HS_ were significantly different between the HT and HL_2arc_, and HL_2arc_ and HL_4arc_ plans (*p* < 0.05). However, the HL_4arc_ (0.40 (IQR: 0.36, 0.42) and 2.62 (IQR: 2.46, 2.85)) plan had the lowest median V_HS_ and V_IS_ for the P_CTV, followed by the HL_2arc_ (0.43 (IQR: 0.39, 0.55) and 2.79 (IQR: 2.56, 3.10)) and HT (0.61 (IQR: 0.56, 0.71) and 3.75 (IQR: 3.40, 4.24)) plans, and significant differences were observed in pairwise comparisons (*p* < 0.05). The median V_LS_ for the P_CTV showed a more pronounced pattern between HT and the HL_4arc_ and HL_2arc_ (*p* < 0.05), except no significant difference was observed between the HL_4arc_ and HL_2arc_ plans in a pairwise comparison (*p* > 0.05). This was well represented in the dose distribution for a patient (Fig. 1). Intermediate and low dose regions (40–65% of the prescribed dose) were well fit to the target volume in HL_4arc_, compared to the HT and HL_2arc_ plans.

No significant differences in NTCPs for the esophagus, spinal cord, and brain stem were observed between the three plans (Table 4). The HL_4arc_ (22.45 (IQR: 18.87, 27.87)) plan had the lowest median NTCP in the parotid glands, followed by the HT (24.82 (IQR: 20.15, 29.66)) and HL_2arc_ (25.92 (IQR: 21.11, 33.71)) plans. In pairwise comparison, the HL_4arc_ plan showed a significant difference median NTCP for parotid glands compared with the HT and HL_2arc_ plan (*p* < 0.05), but no significant difference was observed between the HT and HL_2arc_ plans (*p* > 0.05). The HL_2arc_ (4.48 (IQR: 1.63, 12.80) plan had the lowest median NTCP in the OC, followed by the HL_4arc_ (5.06 (IQR: 1.74, 9.06) and HT (6.18 (IQR: 2.81, 11.15) plans. Furthermore, the HL_4arc_ plan was significantly different compared to the HT plan in the median incidence of NTCP for the OC in pairwise comparison (*p* < 0.05), but no significant difference was observed between the HL_4arc_ and HL_2arc**,**_ and HL_2arc_ and HT plans (*p* > 0.05).

**Table 4:** Comparison of normal tissue complication

Parameters	HT	HL_2arc_	HL_4arc_	*P*^a^		
	Median (IQR)			HT vs. HL_2arc_	HT vs. HL_4arc_	HL_2arc_ vs. HL_4arc_
Esophagus	0.00 (0.00, 0.00)	0.00 (0.00, 0.00)	0.00 (0.00, 0.00)	–	–	–
Parotid glands	24.82 (20.15, 29.66)	25.92 (21.11, 33.71)	22.45 (18.87, 27.87)	0.24	0.02	0.00
Oral cavity	6.18 (2.81, 11.15)	4.48 (1.63, 12.80)	5.06 (1.74, 9.06)	> 0.99	0.01	0.13
Spinal cord	0.00 (0.00, 0.00)	0.00 (0.00, 0.00)	0.00 (0.00, 0.00)	0.13	0.54	0.08
Brain Stem	0.00 (0.00, 0.00)	0.00 (0.00, 0.00)	0.00 (0.00, 0.00)	0.68	0.08	> 0.99

HT, helical tomotherapy; HL_2arc_, halcyon two arc; HL_4arc_, halcyon four arc; IQR, interquartile range (Q1, Q3)

^a^The Wilcoxon signed rank test was used by the Bonferroni correction for multiple testing

## Discussion

Efforts have been made to reduce the dose to OARs near the target volume as much as possible to reduce radiation-induced toxicity and provide a better quality of life during and after RT for NPCa. HT, which was introduced relatively earlier, showed favorable outcomes [18], and FOL was implemented relatively recently for the same purposes in clinical practice. FOL and HT provide similar VMATs based on the same 6 megavoltage photon beam with the FFF design [12, 16] but have distinctly different beam delivery techniques. Various dosimetric characteristics, including dose optimization and calculation algorithm, characteristics of the MLC [32], beam model condition, beam delivery technique etc., interact simultaneously to meet the dose constraints during the invers-dose optimization [35]. Therefore, it is important to evaluate the dosimetric profile of the treatment plan by a new treatment machine in selecting the optimal treatment techniques in order to achieve better clinical outcomes. It is, however, not easy to distinguish which parameters influenced a certain dosimetric profile particularly. This is a plan comparison study, comparing the overall plan quality, based on a typical clinical environment.

Low transmissions of the MLC and interleaf leakage can be effective in reducing normal organ dose outside the target volume during dose optimization based on intensity modulation. It can be considered as one of the many possibilities that contributed to lowering the V_IS_ and V_LS_ for P_CTV in both HL_2arc_ and HL_4arc_ plans, compared to HT in our study. In contrast, Li et al. reported that DL-MLC of the Halcyon™ had no significant impact on plan quality of the head and neck VMAT compared to conventional MLC of Truebeam® (Varian Medical Systems, Palo Alto, CA, USA). It was only effective in IMRT [14].

For OARs sparing, the D_mean_ of the parotid glands was the lowest in the following order—HL_4arc_, HT, and HL_2arc_. However, it doesn’t seem to have clinical significant because both HL_4arc_ and HT met the dose threshold ($\approx 25\mathrm{ Gy}$ for both glands) for severe xerostomia (long-term salivary function < 25%) [7]. High OC dose leads to radiation-induced acute and late toxicity associated with mucositis include oral pain, dysphagia, weight loss, and secondary infections [36, 37]. All plans met the dose threshold for grade $\ge$ 3 acute toxicity (V_30_ > 71.8%) [9] and grade $\ge$ 2 (D_mean_ $\ge$ 50 Gy) [38]. Although the HT plan showed lower D_mean_, V_15_, and V_30_ than both HL_2arc_ and HL_4arc_, NTCP was slightly higher than that of HL_2arc_ and HL_4arc_ plans. This is because the volume receiving high dose (V_45_), which contributes more to NTCP calculation, is relatively larger than these plans. As a result, the differences in NTCP values for parotid glands and OC between the three plans were within 3.47% and 1.7% points, respectively. Furthermore, most of the OARs met the dose criteria in all plans. Although there were slight dose differences depending on the specific plan technique, it does not seem to be clinically meaningful.

When evaluating a new VMAT plan technique, the number of arcs used is an important concern because it has a close relationship with plan quality as well as planning time and beam delivery efficiency. Multiple-arc VMAT plans are likely to provide better dosimetric profile than single-arc VMAT plans for complex targets, with increased delivery times and spread of low doses [10]. However, HL_4arc_ showed better dosimetric profiles than HL_2arc_ but no significant MU increase (median total MU: 836.30 (IQR: 733.15, 918.90) (HL_2arc_) vs. 856.35 (IQR: 750.00, 963.83) (HL_4arc_)) in our study (*p* > 0.05). Also, four arc beams were sufficient to meet the dose constraints. Michiels et al. reported similar results. Triple-arc VMAT plan on a Halcyon provides better dosimeric profile compare with double-arc VMAT plan, and beam delivery time was slightly increased, but significantly decreased compared to double-arc VMAT on a Truebeam [22]. Although more research is needed to determine how many arcs are appropriate for NPCa, FOL provides a greater opportunity to use the multiple-arc VMAT technique because of extremely short beam delivery time, because of fast gantry rotation (four vs. one revolution per minute compared with C-arm Linac) [14].

Although we tried to make a fair and quantitative plan comparison, our study has some limitations. The results of this study may reflect not only the differences in the intrinsic performance of the treatment machine and beam delivery technique, but also the differences in dose calculation grid resolution, dose calculation error, and dose optimization capability of the treatment planning system and planning skill of the dosimetrists between the two comparison groups. It seems necessary to consider them when referring to the results of this study.

## Conclusions

With the advancement of RT technology, new treatment machines and beam delivery techniques are being introduced steadily to meet clinical demands for the improvement of clinical outcomes and reduction of radiation-induced complications. It is timely and important to have a clear understanding of the capabilities and limitations of a new device prior to patient assignment in clinical practice.

We performed the first dosimetric comparison study between VMAT plans using the FOL with DL-MLC and HT with dynamic jaw for NPCa. Although HT and FOL plans showed different advantages and disadvantages in dosimetric characteristics, overall, they showed comparable dosimetric profiles. Our results, combined with the facility’s availability of treatment machines and resource, may contribute to wise selection of appropriate RT techniques for NPCa.

## Supplementary Information

** Additional file 1.** The parameters used for NTCP evaluation.

## Data Availability

The datasets analyzed during the current study are available from the corresponding author on reasonable request.
