# Supplementary material for: Dosimetric comparison between VMAT plans using the fast-rotating O-ring linac with dual-layer stacked MLC and helical tomotherapy for nasopharyngeal carcinoma
Source: Radiat Oncol. 2022 Sep 12;17:155. doi: 10.1186/s13014-022-02124-0 (PMC9465858; doi:10.1186/s13014-022-02124-0)
Supplement: Supplementary file 1 — Additional file 1. The parameters used for NTCP evaluation. [file 13014_2022_2124_MOESM1_ESM.docx]

Supplementary table 1:

| **Description** | **Endpoint** | **D50** | **Gamma** | **α/β** | **Seriality** |
| --- | --- | --- | --- | --- | --- |
| Esophagus | Clinical stricture/perforation | 68.4 | 6.55 | 3 | 0.22 |
| Parotid | xerostomia | 46 | 1.8 | 3 | 1 |
| Oral cavity | Mucositis | 39 | 3 | 3 | 0.5 |
| Spinal cord | myelitis | 68.6 | 1.9 | 3 | 4 |
| Brain Stem | necrosis | 65.1 | 2.4 | 3 | 1 |
